# *De novo* Synthesis of Glial Glutamate and Glutamine in Young Mice Requires Aspartate Provided by the Neuronal Mitochondrial Aspartate-Glutamate Carrier Aralar/AGC1

**DOI:** 10.3389/fendo.2013.00149

**Published:** 2013-10-15

**Authors:** Beatriz Pardo, Laura Contreras, Jorgina Satrústegui

**Affiliations:** ^1^Departamento de Biología Molecular, Centro de Biología Molecular Severo Ochoa UAM-CSIC, CIBER de Enfermedades Raras (CIBERER), Universidad Autónoma de Madrid, Madrid, Spain

**Keywords:** AGC1, aralar, aspartate, glial glutamine, mitochondrial aspartate-glutamate carrier

## Introduction

In brain, the glutamate-glutamine and the glutamate-glutamine-GABA cycles are essential for efficient glutamatergic and GABAergic neurotransmission. The interactions between neurons and astrocytes required for the operation of these cycles have received considerable attention since their discovery (1). In gray matter, glutamate released from glutamatergic neurons is mostly taken up into astrocytes (2, 3) where it is converted into glutamine which is sent back to the neurons for conversion to glutamate. This drain of glutamate released from neurons to astrocytes is compensated for by a flow of glutamine from astrocytes to neurons, thus closing the glutamate-glutamine cycle.

In addition to transcellular cycling, the glutamate-glutamine cycle faces losses of components, mainly glutamate, by oxidation, which is balanced by the anaplerotic synthesis of glutamate and glutamine in astrocytes. As shown by Rothman (4) and discussed by Hertz (5), *in vivo* about 85% of the glutamate cycles in the glutamate-glutamine cycle but 15% is oxidized. Oxidation may take place in astrocytes and neurons, and under basal conditions it does not appear to require glutamate dehydrogenase (GDH), a mitochondrial enzyme present mainly in astrocytes, as a brain-specific-disruption of the gene has small effects on brain glutamate levels (6). *De novo* synthesis of glutamate takes place in astrocytes thanks to pyruvate carboxylase (PC) (7, 8), which is expressed in astrocytes but not in neurons (9–11). This process generates a new molecule of oxaloacetate, which may condense with acetyl-CoA to provide net synthesis of α-ketoglutarate, from which glutamate can be formed by transamination (12). Subsequently, glutamine will be synthesized from glutamate via glutamine synthetase, which is exclusively present in the cytosol of astrocytes (13, 14). However, this general picture of the glutamate-glutamine cycle may be oversimplified, and there may be important variations, at least, during postnatal development. Recent metabolic studies in the neonatal rat brain have shown that the flow of glutamate from neurons to astrocytes is negligible when compared to the adult brain (15), while, paradoxically, the flow of glutamine from astrocytes to neurons is the same or even larger than in adults. This may suggest that astrocytes are slowly supplying glutamine to neurons so as to build up the neuronal pools of glutamate, aspartate, and N-acetylaspartate (NAA), which are lower than in the adult brain (15), and also, that a molecule other than glutamate is being transferred from neurons to astrocytes.

## Brain Aspartate, N-Acetylaspartate, Glutamine and Glutamate Levels, and the Synthesis of Glutamine *in vivo* are Reduced in Aralar-Deficient Mice

Aralar/AGC1/Slc2512 is an isoform of the mitochondrial carrier of aspartate-glutamate (AGC), a component of the malate-aspartate shuttle (MAS) and it is regulated by calcium from the external side of the inner mitochondrial membrane (16). MAS activity allows the reoxidation of cytosolic NADH which is required to maintain glycolysis coupled to pyruvate oxidation in mitochondria rather than lactate formation. Cytosolic calcium sensed by EF-hands in the N-terminal extensions of aralar carrier regulates MAS activity in neurons (17) and β-cells (18) and Ca^2+^-activated respiration in intact neurons (19).

The deficiency in aralar results in a drastic fall in brain aspartate and NAA levels, in a fall in NAA production by neurons in culture, and global hypomyelination both in mice (20–22) and humans (23). Hypomyelination has been attributed to a lack of neuronal NAA which, after transaxonal transport and cleavage by aspartoacylase, is required to supply acetyl groups to developing oligodendrocytes for myelin lipid synthesis (20, 24), energy metabolism (25), or other essential functions [reviewed in Ref. (22)].

Aralar deficiency also results in a large fall in brain glutamine content but only a modest decrease in brain glutamate levels, which is not associated to decreases in neuronal glutamate content or changes in synaptosomal glutamate release (26). None of the activities of the three main enzymes involved in the glutamine metabolism [phosphate-activated glutaminase, GDH, and mitochondrial aspartate aminotransferase (mAST)] are changed in the brain of the aralar-KO mice (26). The synthesis of glutamate and glutamine *in vivo* was also analyzed in aralar-deficient mice at P18-20 after labeling with (1,2-^13^C)acetate or (1-^13^C)glucose. Interestingly, labeled glutamine in the brain of aralar-KO mice was found to be below detection limit, regardless of whether (^13^C_2_)acetate or (1-^13^C)glucose were used as cerebral substrates. These results clearly pointed out to a role for neuronal aralar in glial glutamine production in mouse brain.

## Expression and Functional Relevance of Aralar in Brain Cells

A decrease in brain glutamine synthesis in aralar-KO mice is puzzling, in the face of the prominent expression of aralar in neurons rather than in brain astrocytes (26–30). This preferential expression in neurons was surprising, as aralar, and also citrin/AGC2/Slc25a13, is definitely present in cultured astroglia (26, 27, 31) and aralar mRNA has been reported to be present in brain astrocytes (31, 32). However, whether aralar is actually used in astrocytes in culture is uncertain, as the lack of aralar in cultured astrocytes did not cause the metabolic alterations characteristic of its deficiency in neurons [increased utilization of external pyruvate, decrease in aspartate levels; (26)]. So far, the most accurate determination of the localization of aralar in brain was that obtained by electron microscopy and immunogold-particle labeling of neuronal and astrocytic mitochondria, especially as the specificity of the labeling was contrasted with the use of aralar-KO mouse brains. These studies showed that neurons contained 94% of the labeled profiles whereas glial cells (both astrocytes and oligodendrocytes) contained only about 7%, close to the background level (26). These data clearly indicate that, at the protein level, aralar is localized preferentially, if not exclusively, in neurons.

## Role of Aspartate in Glutamine Synthesis by Astrocytes

The most striking defect in the aralar-deficient neuron is the complete loss of aspartate. In addition, the levels of other amino acids which give rise to pyruvate also decrease, in response to a failure of MAS which results in a limitation in pyruvate supply to mitochondria. As the defect in glutamine synthesis is necessarily astrocytic, a metabolic limitation in the synthesis of glutamate and glutamine was suspected to happen in brain astrocytes. As the levels of α-KG were also maintained in the KO brains, the drop in glial glutamate-glutamine synthesis was attributed to a shortage of the amino-group donor (26). It is known that the *de novo* glutamate and glutamine production in astrocytes requires the supply of one or two ammonia groups, respectively, and neurons are thought to supply one or both (33). This would depend on whether glutamate oxidation occurs in astrocytes, which would maintain the amino group of glutamate in the astroglial cell, or in neurons, which would not. In this case, neurons would need to supply two amino groups to neighboring astrocytes per newly made glutamine.

It is currently believed that most of the glutamate oxidation occurs in astrocytes (4). Indeed, photoreceptors in the retina avoid the oxidation of glutamate through a tight regulation of the fate of α-KG exerted by the NAD^+^/NADH ratio (34). However, glutamate is also oxidized in photoreceptors and neurons. For example, under hypoglycemia glutamate oxidation takes place through the truncated tricarboxylic acid cycle and aspartate becomes the end product (35–38). The relative importance of astrocytes versus neurons in the oxidation of glutamate in the intact brain both at rest and in conditions of activation is still unknown.

Astrocytes take up aspartate through the same plasma membrane transporters that take up glutamate and with the same affinity (39). In astrocytes, ^15^N-aspartate is rapidly taken up from the medium (40, 41), and its amino group is transferred to glutamate in the aspartate aminotransferase reaction, which then gives rise to glutamine. The fate of the carbon skeleton of aspartate in astrocytes has been also addressed in metabolic studies with U-^13^C-aspartate. The results indicate that after an initial transamination, to give rise to oxaloacetate and glutamate, oxaloacetate enters the TCA and is largely incorporated into glutamine. Another large fraction of oxaloacetate is converted into lactate through pyruvate recycling (42–44). These facts suggested that aspartate produced by neurons may be required for glutamate and glutamine production in astrocytes, at least during postnatal development.

**Figure 1:**
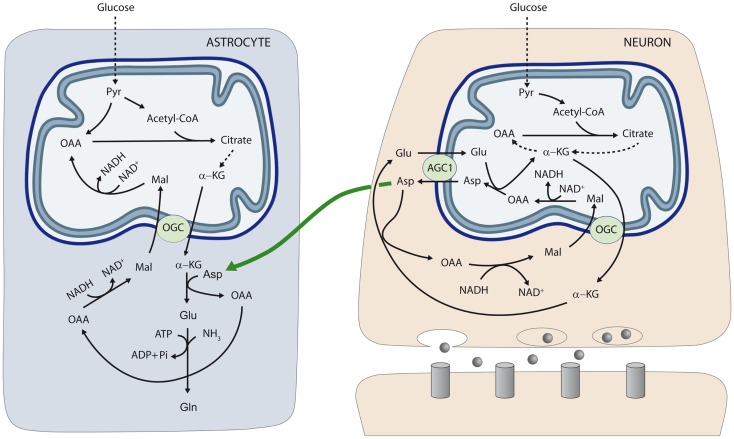
**Neuron-to-glia transcellular aspartate efflux pathway for glial glutamate synthesis**. Neuronal mitochondria are provided with aralar/AGC1/Slc25a12 and the oxoglutarate carrier/OGC/Slc25a11 and carry out the malate-aspartate shuttle to transfer NADH reducing equivalents to the mitochondrial matrix. AGC1 is irreversible in polarized mitochondria and the main pathway of glutamate supply to the mitochondrial matrix. As cAST functions in the direction of glutamate formation in cells with an active MAS, mitochondria are the only site where aspartate is produced (in the mitochondrial aspartate aminotransferase reaction), and aspartate leaves the matrix through AGC1 to reach the cytosol. *De novo* glutamate synthesis in astroglial cells takes place in the cytosol in the cAST reaction with aspartate as amino-nitrogen donor to α-KG. A second amino group (possibly arising from ammonia itself formed in neurons in the phosphate-activated glutaminase reaction, or imported from the blood stream) is acquired in the glutamine synthetase reaction and glial glutamine is now transferred to neurons along the glutamate–glutamine cycle (not shown). Oxaloacetate (OAA) arising from the cAST reaction is converted to malate, and malate entry in glial mitochondria along the OGC provides an alternative pathway for redox transfer to mitochondria, which partly compensates for the lack of a malate-aspartate shuttle in brain astrocytes. In this way, equivalent transfer to astroglial mitochondria is stoichiometrically related to *de novo* glutamate production. Alternatively, malate formed in astroglial cytosol may be transferred back to neurons, as malate is released to a higher extent from cultured astrocytes than from cultured neurons (45) (not shown). The diagram does not address the regulation of the two fates of aspartate in the neuron, MAS (as depicted) or the transfer to the astrocyte (as also depicted), which are obviously mutually exclusive. Thus, transfer of aspartate to astrocytes is associated with glutamate oxidation in neurons, possibly through a truncated TCA cycle, rather than the operation of MAS. AGC, aspartate–glutamate carrier; Asp, aspartate; Gln, glutamine; Glu, glutamate; α-KG, α-ketoglutarate; Mal, malate; OAA, oxaloacetic acid; OGC, α-ketoglutarate–malate carrier; Pyr, pyruvate. Gray circles at presynaptic neuron represent neurotransmitter released; and gray columns at postsynaptic neuron the corresponding receptors [reproduced from Ref. (26)].

To test the role of aspartate in astrocytic glutamate and glutamine synthesis, astrocytes were incubated in the presence of glucose and different amino acids. The supply of 50–200 μM aspartate during 1 h to astroglial cultures resulted in glutamate and glutamine levels significantly higher than those obtained with all the other amino acids tested including alanine, BCAAs (leucine), and GABA, which, in fact, did not enhance glutamate or glutamine synthesis. Of note, this effect of external aspartate was identical in astrocytes derived from wild type or aralar-KO mice. These findings showed that aspartate is the main amino-group donor for *de novo* glutamate synthesis in astrocytes and explain why the lack of aspartate produced in neurons causes an impairment in the synthesis of glutamine in aralar-KO mice (26). On the basis of these results we proposed a transcellular traffic of aspartate from neurons to astrocytes which is summarized in Figure 1.

l-Aspartate was proposed to act as a neurotransmitter (46), but this hypothesis has been largely abandoned. However l-aspartate can be taken up in synaptic vesicles and exocytotically released form nerve terminals (47). Whether this is the mechanism utilized for the transcellular flux of aspartate from neurons is unknown. Another possibility for the traffic of aspartate from neurons to astrocytes may involve NAA, as the neuronal transporters of NAA are well established, as is its uptake by astrocytes and subsequent cleavage by aspartoacylase to release acetate and aspartate [reviewed in Ref. (22)].

The neuron-to-astrocyte aspartate efflux pathway described in Figure 1 may provide a means to transfer NADH/NAD^+^ redox potential to astrocyte mitochondria an alternative transcellular shuttle system. Indeed, aspartate uptake coupled to OAA production provides a substrate for cytosolic malate dehydrogenase resulting in NADH consumption in the cytosol and malate formation. As the α-ketoglutarate–malate carrier is equally represented in neuronal and glial mitochondria (28), aspartate utilization in glutamate formation in astrocytes will be stoichiometrically related to reducing equivalent transfer to mitochondria. In this way, transcellular aspartate traffic would result in malate oxidation by astrocytic mitochondria. Malate formed in astroglial cytosol may be also transferred back to neurons, as malate is released to a higher extent from cultured astrocytes than from cultured neurons (45).

Alternatively, Hertz (5, 48) has suggested that aralar/AGC1 in brain astrocytes, even at very low levels, could play a role in a modified aspartate-malate shuttle to oxidize reducing equivalents in mitochondria. In this modified shuttle OGC and AGC1 are involved in two different functions: the OGC, in glutamate formation (as shown in Figure 1) and the AGC1 in glutamate oxidation.

## Concluding Remarks

The role of neuronal aspartate as precursor of glutamate synthesis in astrocytes explains the alterations found in the brains of aralar/AGC1 KO mice, specifically, the impressive drop in the synthesis of glutamine. However, these findings were obtained at 18–20 days postnatal, at a time where the full development of the glutamate/glutamine cycle has not yet taken place. Further studies in adults will be required to fully understand the role of this transcellular pathway.
